# Enabling Low-Latency Bluetooth Low Energy on Energy Harvesting Batteryless Devices Using Wake-Up Radios

**DOI:** 10.3390/s20185196

**Published:** 2020-09-12

**Authors:** Ashish Kumar Sultania, Carmen Delgado, Jeroen Famaey

**Affiliations:** IDLab-Department of Computer Science, University of Antwerp-imec, 2000 Antwerp, Belgium; carmen.delgado@uantwerpen.be (C.D.); jeroen.famaey@uantwerpen.be (J.F.)

**Keywords:** Bluetooth Low Energy (BLE), wake-up radio (WuR), friendship, poll, batteryless, Low Power Node (LPN)

## Abstract

With the growth of the number of IoT devices, the need for changing batteries is becoming cumbersome and has a significant environmental impact. Therefore, batteryless and maintenance-free IoT solutions have emerged, where energy is harvested from the ambient environment. Energy harvesting is relevant mainly for the devices that have a low energy consumption in the range of thousands of micro-watts. Bluetooth Low Energy (BLE) is one of the most popular technologies and is highly suitable for such batteryless energy harvesting devices. Specifically, the BLE friendship feature allows a Low Power Node (LPN) to sleep most of the time. An associated friend node (FN) temporarily stores the LPN’s incoming data packets. The LPN wakes up and polls periodically to its FN retrieving the stored data. Unfortunately, the LPNs typically experience high downlink (DL) latency. To resolve the latency issue, we propose combining the batteryless LPN with a secondary ultra-low-power wake-up radio (WuR), which enables it to always listen for an incoming wake-up signal (WuS). The WuR allows the FN to notify the LPN when new DL data is available by sending a WuS. This removes the need for frequent polling by the LPN, and thus saves the little valuable energy available to the batteryless LPN. In this article, we compare the standard BLE duty-cycle based polling and WuR-based data communication between an FN and a batteryless energy-harvesting LPN. This study allows optimising the LPN configuration (such as capacitor size, polling interval) based on the packet arrival rate, desired packet delivery ratio and DL latency at different harvesting powers. The result shows that WuR-based communication performs best for high harvesting power (400 μW and above) and supports Poisson packet arrival rates as low as 1 s with maximum PDR using a capacitor of 50 mF or more.

## 1. Introduction

**Figure 1:**
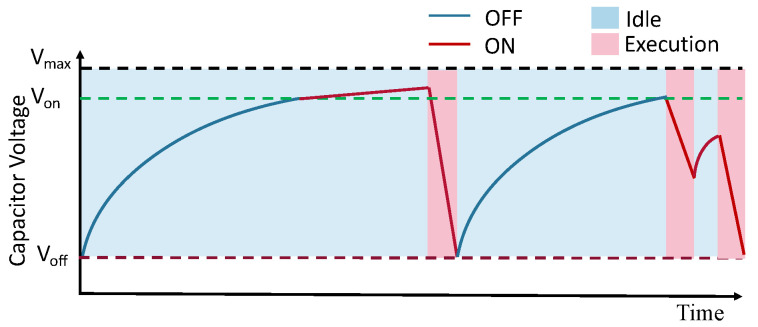
Intermittent behaviour of capacitor enabled device harvesting continuously.

The Internet of Things (IoT) has managed to make the world a connected place with a growing number of devices. It is projected that by 2022 the Internet will connect 14.6 billion IoT devices [1]. With this growth, there is a need to maximize their energy efficiency to maximize device lifetime. Usually, these devices use batteries as the primary energy source. However, battery replacement for such a large number of IoT devices is not only impractical but also impacts the environment due to the harmful chemicals that discarded batteries can leak into the soil. To solve this problem, environment-friendly capacitors and energy harvesters can replace the batteries. Such batteryless devices have applications in tracking goods in warehouse logistics, monitoring environmental conditions, wild-life monitoring and can be installed as in-body devices. Capacitors support a vast number of charging cycles and thus have a much longer lifetime than batteries. However, the much smaller energy density of the capacitor and unpredictable availability of harvested energy result in intermittent on-off behaviour of the device, as shown in Figure 1. When the capacitor voltage drops below the minimum operating voltage ($V_{off}$), the device will turn off. Therefore, the device needs an energy harvester to replenish the energy stored in its capacitor continuously. A variety of environmental sources, such as light and motion, can be used to harvest energy. As shown in Figure 1, after the device turns off, it will turn on again only when achieving a predefined turn-on voltage $V_{on}$.

Bluetooth Low Energy (BLE), being a short-range energy-efficient communications technology is highly suitable for batteryless devices [2]. The BLE specification [3] already provides a friendship feature to ease its Low Power Nodes (LPNs) to save energy keeping themselves in sleep mode or turned off most of the time. LPNs awake only to transmit or receive data packets. For sending uplink (UL) data packets, the LPN can broadcast them at any time. The LPN can start a poll process (request/response) to receive the downlink (DL) data that are temporarily buffered at the friend node (FN). The polling happens periodically (based on a predefined duty-cycle) to receive any incoming data packets. This can reduce the overall energy consumption but increases the DL latency. Additionally, the duty-cycled polling can also lead to wasted energy by sending and receiving poll requests/responses when no data is buffered. Therefore, to reduce the DL latency and superfluous polling, it is required to align the LPNs polling with the moment when the FN receives incoming packets. Such coordination can be accomplished by an additional secondary wake-up radio (WuR).

**Figure 2:**
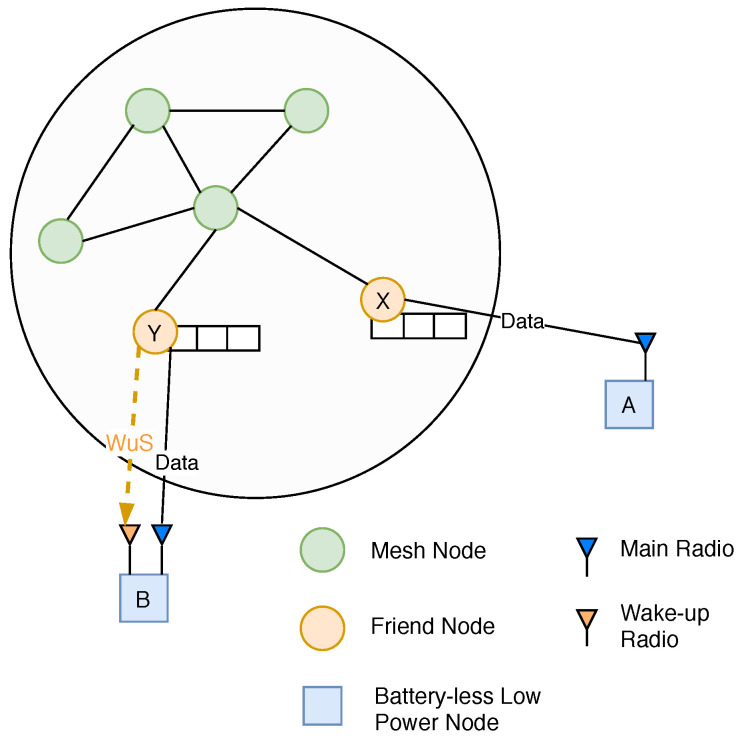
Overview of Bluetooth Low Energy (BLE) mesh network with integrated batteryless Low Power Nodes (LPNs), both with and without wake-up radio (WuR) support.

As the WuR’s power consumption (few tens of μW) is many orders of magnitude lower than the main radio’s (hundreds of mW), it can be kept in listening mode (switching the main radio to sleep mode) even when the device is powered by harvested energy [4,5]. This allows the FN to notify the LPN when a DL packet is available by sending a wake-up signal (WuS). The reception of a WuS then triggers the LPN to start the standard friendship polling process to receive the DL data. As shown in Figure 2, there can be two types of batteryless LPNs. LPN-A connected with FN-X is the standard node without WuR. LPN-B is WuR-based and can receive the WuS from its associated FN-Y. An FN can support friendship with a maximum of seven LPNs (any types) simultaneously. Each FN maintains multiple buffers to store its corresponding LPN’s data packets.

A small amount of additional power consumption due to WuR can have a significant effect on the energy availability in a device which has a harvesting power in the order of tens to hundreds of μW. To the best of our knowledge, this work is the first to investigate the network performance and requirements for a batteryless LPN in combination with a WuR. It is required to know the optimal capacitance for distinct harvesting powers at which the LPN can perform its operations without experiencing an outage. In this article, we evaluate the optimal capacitor size and polling interval for an LPN at different harvesting powers, by maximising packet delivery ratio (PDR) and minimising DL latency. We also evaluate the possible benefits of integrating a WuR in the LPN and compare the system with the standard polling approach.

The outline of this article is as follows. In Section 2, we provide an overview of the related literature. In Section 3, we present an introduction to the friendship feature explaining the communication between the LPN and the FN. The system considers both the types of LPNs, the standard one without WuR and the modified LPN enabled with WuR are considered in the discussion. Additionally, a model of the batteryless device is presented to calculate its voltage over time. The system performance is analysed in Section 4, and Section 5 presents the conclusion and future work.

## 2. Related Work

Energy harvesting has been extensively explored to support sustainable operations of IoT systems. Various types of ambient energy have been utilized, such as radio frequency (RF), solar and wind energy. An overview of the energy harvesting technologies for various applications is presented in [6,7,8]. Meli et al. [9,10] demonstrated the suitability of the BLE protocol for a battery-free IoT device. They concluded that it is possible to use the BLE wireless standard in combination with energy harvesters. They showed battery-free BLE devices powered with solar cells in a room or building environment could broadcast pre-programmed information such as GPS coordinates. Batteryless BLE prototypes for smart building applications have also been presented, making use of ambient light energy [11] or RF energy harvesting [12]. Sanislav et al. [13] implemented a proof-of-concept design of a BLE device based on a wireless energy harvesting element. The node, equipped with a 50 mF capacitor, is charged by an RF energy harvester module harvesting from a 5 m apart GSM mobile. It can take measurements once every 30 s. Brunecker et al. achieved up to 32 mW harvested power using a 6 dBi gain transmitter antenna placed at 5 cm from the harvesting receiver and up to 1.5 mW at 40 cm distance [14]. Zhong et al. [15] implemented their design of an implantable batteryless bladder pressure monitor system that monitors bladder storage in real-time and transmits the feedback signal to the external receiver through BLE. They use a four-coil wireless energy transmission method, which supports a power transmission range of up to 7 cm. Another solution is having a batteryless BLE beacon powered by a customized water leak sensor as proposed by Witham et al. [16]. They considered a peak short-circuit harvesting current of 8.1 mA and a peak current requirement of 8.25 mA during radio events of the BLE beacon. So, a capacitor of 3.9 mF enabled the BLE beacon to transmit the data for a short time. All the mentioned researchers focus only on the UL data. In contrast, our work studies the ability for a batteryless device to receive DL data using the friendship feature. We also focus on evaluating the optimal capacitor size required to receive data packets for different harvesting power ranges. A control loop system is proposed where the nodes can request the energy source to provide energy enabling them to replenish their storage (capacitor or battery) [17]. This method can ensure a certain level of quality-of-service for an IoT application. They also present a prototype using BLE nodes equipped with a photovoltaic energy harvester, which communicate and request a recharge from an indoor smart lighting system.

The detailed surveys on WuR hardware and protocols are discussed in [18,19]. Many researchers presented the design to couple a low power WuR with BLE to explore its potential. Many WuRs are also implemented to be triggered using BLE packets [20,21,22]. Giovanelli et al. [23] evaluated the possible benefits of integrating a WuR in the BLE protocol stack. They observed that the use of a WuR reduces energy consumption and DL latency. The WuR decreases the DL latency by up to 40% in the case of connection-oriented communication when the number of devices is large (100+), while with few devices, the traditional approach performs better. Mikhaylov et al. [24] demonstrated that the WuR-based BLE can outperform the classic BLE solution (without WuR) if the maximum latency for data delivery tolerable by the application does not exceed 2.1 s. Sanchez [25] also evaluated that WuR-based BLE performs better than the classic BLE for low frequent data rates. They performed the tests for battery-powered nodes, whereas our work focuses on batteryless nodes. This is expected to impact the results and conclusions significantly. Specifically, the low harvesting power density combined with the added power consumption of a WuR can worsen the intermittent behaviour of a batteryless device. Other works that have integrated the WuR capabilities in BLE are reported in [21,26,27]. These works focused on hardware design aspects of WuR-integration, while our work looks at protocol aspects instead. Liu et al. [28] presented an RF-based passive WuR enabled batteryless node. They observed that the energy harvested within 100 ms at a distance of 1 m from the RF energy transmitter is sufficient to transmit and receive 40 B long beacon messages in a range of 3 m. Whereas they target a fixed type of harvesting technology without considering the impact of the capacitor, this article investigates the optimal size of a capacitor for different ranges of harvested power. At the time of writing, no work presents the evaluation of the BLE friendship feature considering batteryless LPNs. As such, this work is the first to study the DL performance of batteryless LPNs. Moreover, we consider combining the batteryless device with a WuR to optimize the DL latency further.

## 3. Batteryless LPN Design

In this section, the communication between the FN and the batteryless LPN is described. First, we summarize the friendship feature of the Bluetooth mesh specification [3] and introduce the modification required in the communication mechanism by adding a WuR in an LPN to reduce the DL latency. Next, we present the batteryless LPN model to calculate its available capacitor voltage over time, as a function of the energy harvesting power, capacitance, and energy consumption. Lastly, we explain the behaviour of a batteryless LPN and usage of the model to predict the time when to perform communication with its FN. Both the communication schemes are described one when the LPN polls directly using the main radio and another when it has a WuR.

### 3.1. BLE Friendship Feature

The devices that join a Bluetooth mesh network are called nodes. They follow a publish-subscribe communication pattern. A node publishes messages to send, and receivers can subscribe to the sender’s address to receive them. The nodes can possess optional additional features based on their capabilities in the network. These features categorize the nodes as a relay, proxy, friend, or low power node. The relay nodes support the re-transmission of data packets that are broadcast by other nodes. They help in extending the range of the entire network. The proxy nodes help the non-mesh-supported BLE devices to communicate via the mesh network. The FN and LPN have a friendship relationship where the FN receives and stores the DL data packets intended for the associated LPN while the LPN sleeps or temporarily shuts down. The FN maintains multiple friend queues (FQs), one for each connected LPN, to store all the incoming data packets [29]. The maximum size of an FQ containing 16 bytes of lower transport protocol data units (PDUs) that the LPN can request is 128 packets. The data can be retrieved later by the LPNs using a polling mechanism. This provides the LPNs with the flexibility to remain mostly in the lowest power state. A node in the network can enable or disable its responsibility to support these four features, as they come with an additional overhead while connected to the network.

**Figure 3:**
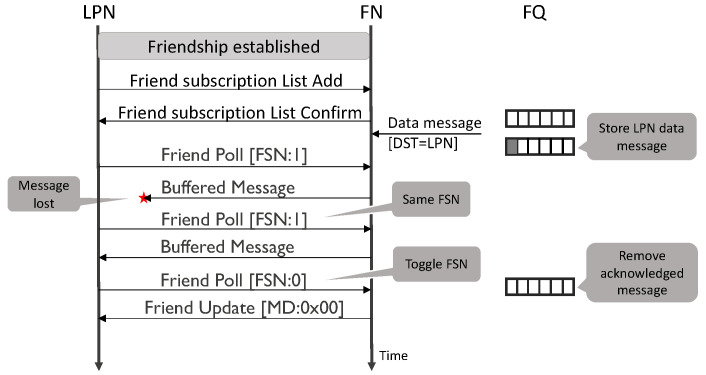
Network messages between the LPN and friend node (FN).

According to the specification, the LPN initiates the request to establish a friendship relationship. The neighbouring nodes (within a single hop) can respond for the acceptance with their capabilities to become a friend. Subsequently, the LPN accepts one of the best capable nodes to be its friend. A node cannot have the low power feature enabled unless a neighbouring node agrees to be a friend. Additionally, an FN needs a sustainable power supply to keep it awake consistently. An FN can support friendship with a maximum of seven LPNs simultaneously. Whereas, an LPN can be a friend with only one FN. Figure 3 shows an example of the message exchange between the LPN and FN after establishing the friendship. The LPN sends a friend subscription list message to the FN, which contains all its subscribed addresses. This list enables the FN to identify which messages to buffer for the LPN. The LPN periodically sends a friend poll (FP) message to the FN to get any stored data and to keep the connection alive. The FP messages are sent in all the three BLE advertising channels (37–39). After receiving the FP message, the FN replies with the oldest buffered data packet. It discards the packet from the FQ once the LPN acknowledges its reception. The acknowledgement consists of a single bit and is referred to as the friend sequence number (FSN). The LPN toggles the FSN each time it successfully receives a packet. Therefore, the FN sends another entry of the FQ, if it receives an FP message that has a different FSN field value as in the previously received FP message. If the FSN is the same, it retransmits the previous message (if it has not been discarded in the meantime). The FN returns a friend update (FU) message if the FQ is empty or when the security parameters for the network have changed. The FN signals the FQ occupancy to the LPN via the 1-byte More Data (MD) flag in the FU message. If MD equals 0, it denotes the FQ is empty, 1 that it is not. The values 2 to 255 are reserved for future use.

**Figure 4:**
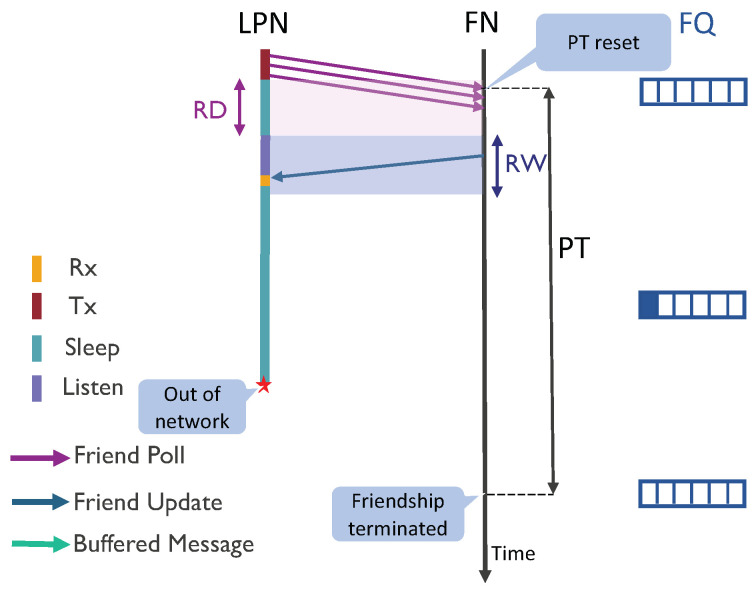
Friendship termination by the FN.

The standard BLE friendship protocol defines three timing parameters: ReceiveDelay (RD), ReceiveWindow (RW), and PollTimeout (PT), that are fixed for a session of the relationship. These timers are negotiated during the process of the friendship establishment. The LPN presents the timer values for RD and PT in the friend request message whereas the FN proposes the RW in the friend offer message. These timers can be seen in Figure 4. The RD and RW can be configured with a maximum value of 255 ms and the PT with a maximum of 96 h. RD is the delay between the LPN sending the FP message and when it starts listening to a response from the FN. During the RD, the LPN can turn off its radio and switch to sleep mode. During RW, the LPN expects the data and listens actively for it. The LPN uses more energy due to active listening during RW, and therefore, a short RW should be configured if possible. The PT is used as a timeout to ensure that when an LPN leaves the network, its friendship relationship is not kept alive indefinitely. If the FN does not receive any poll request from an LPN before the PT timer expires (since the last poll), it terminates the friendship with that LPN and removes the corresponding FQ.

As mentioned above, the data communication is initiated by the LPN based on its duty cycle, and independent of the arrival of DL data packets at the FN. As such, the DL latency can become very high if the LPN has a long duty cycle (which is often desirable to minimize energy consumption). To solve this, adding a WuR to an LPN provides the FN with the ability to notify it in advance about the incoming data packet before the LPN initiates polling. The LPN enabled with a WuR can continuously listen to the incoming WuS, as WuR idle listening power consumption is orders of magnitude lower than that of the main radio. The FN sends a WuS to the LPN whenever it receives a network packet corresponding to that LPN in an empty FQ. The LPN (having sufficient energy) polls until it receives an FU message indicating there are no messages buffered in the FQ. Upon receiving the WuS by the LPN’s WuR, it subsequently triggers the main radio to initiate to send the FP message. This reduces the need for sending frequent periodic FP messages and polls only when data is actually buffered in the FQ. Thus, it not only reduces the DL latency but also reduces the energy wastage due to polling without receiving buffered data.

### 3.2. Batteryless Device Model

**Figure 5:**
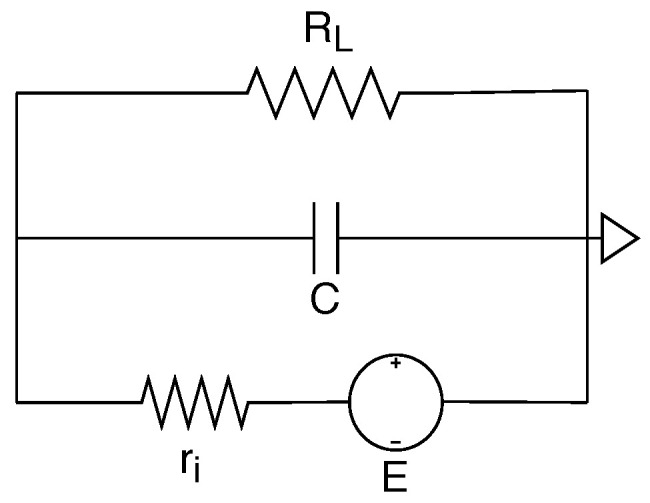
Electrical circuit model of a batteryless IoT device.

A batteryless LPN is equipped with an energy harvester, a capacitor, a micro-controller unit (MCU), a main radio and an optional WuR. The batteryless circuit is introduced by Delgado et al. [30] to calculate the voltage of a batteryless device at a specific time as shown in Figure 5. Assume the device maximum operating voltage is *E* (volt) and the harvester provides a power of $P_{h}$ (watts) modelled as a real voltage source having an internal resistance $r_{i}$ (ohm). In order to limit the power of the harvester, this internal resistance $r_{i}$ is defined as $E^{2}/P_{h}$. The energy-consuming components are the MCU, main radio, WuR, or any other peripherals that are modelled as a load resistance. The current consumption of these components varies with their operating states (e.g., sleep, active). Let the total current consumption of the LPN load at a time instant *t* be $I_{L}\left(t\right)$ (ampere) then according to the Ohm’s law, the load resistance of the LPN $R_{L}\left(t\right)$ = $E/I_{L}\left(t\right)$. As the capacitor, the harvester and the LPN components have a parallel connection, the equivalent resistance of the LPN is calculated as Equation (1).

(1) $$\begin{array}{c} R_{eq}\left(t\right)=\frac{1}{\frac{1}{r_{i}}+\frac{1}{R_{L}\left(t\right)}}=\frac{R_{L}\left(t\right)\cdot r_{i}}{R_{L}\left(t\right)+r_{i}}=\frac{E^{2}}{P_{h}+E\cdot I_{L}\left(t\right)}\;. \end{array}$$

By applying Kirchoff’s voltage law to the circuit, the voltage of across the load *V*(*t*+Δ*t*) over a time period Δ*t* starting at time *t*, given its voltage V(t) at a time *t*, having a capacitor of *C* (farad) and a fixed resistance $R_{eq}\left(\Delta t\right)$ during the time interval Δ*t* can be calculated as Equation (2) [30].

(2) $$\begin{array}{c} V\left(t+\Delta t\right)=E\cdot \frac{R_{eq}\left(\Delta t\right)}{r_{i}}\left(1-e^{\frac{-\Delta t}{R_{eq}\left(\Delta t\right)\cdot C}}\right)+V\left(t\right)\cdot e^{\frac{-\Delta t}{R_{eq}\left(\Delta t\right)\cdot C}}\;, \end{array}$$

After substituting the value of $r_{i}$ and $R_{eq}\left(\Delta t\right)$ (Equation (1)) in Equation (2), the final voltage is derived as Equation (3).

(3) $$\begin{array}{c} V\left(t+\Delta t\right)=\frac{E\cdot P_{h}}{P_{h}+E\cdot I_{L}\left(\Delta t\right)}\left(1-e^{\frac{-\Delta t\cdot (P_{h}+E\cdot I_{L}\left(\Delta t\right))}{E^{2}\cdot C}}\right)+V\left(t\right)\cdot e^{\frac{-\Delta t\cdot (P_{h}+E\cdot I_{L}\left(\Delta t\right))}{E^{2}\cdot C}}\;. \end{array}$$

The formula mentioned in Equation (3) calculates the voltage change of an LPN while its state (and thus the current consumption) remains the same during a time interval Δ*t*. It needs to be recalculated every time the LPN’s state changes.

### 3.3. Poll-Based LPN

**Figure 6:**
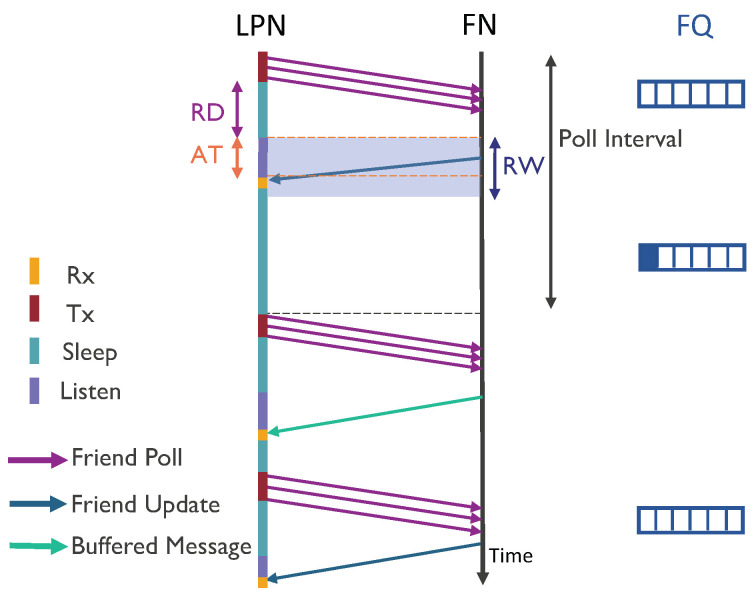
Sequence diagram to receive downlink (DL) data packets using poll-based communication.

In the default BLE friendship mechanism, the LPN initiates the communication by sending an FP message. Figure 6 shows a sequence diagram representing the communication steps. It can be seen that the FP message is sent in the three broadcast channels and then the LPN switches to sleep mode until the RD expires. During the RW timer, the FN starts sending the buffered message, which is received by the LPN after arrival time (AT). So, the LPN stops listening at this point and starts receiving the buffered message. The AT depends on the required processing time at FN, queuing time (e.g., because the FN first needs to send a packet to another node), and propagation delay. As mentioned above, the AT should always be smaller than the RW, as after that time, the LPN stops listening and the response would thus be lost. To avoid receiving frequent, continuous FU messages, a timer poll interval is introduced. On receiving an FU message (meaning that the FQ is empty), an LPN needs to wait until the poll interval timer expires to send the next FP message. The poll interval should be less than the PT, to avoid the FN from disconnecting the LPN. In the sequence diagram, the LPN first receives an FU message because the FQ is empty. So, it waits until the poll interval time expires to send the next FP messages. The FN subsequently replies with a buffered data packet, which is removed from the FQ on receiving the third FP messages. At this point, the FQ is empty again, and the FN thus sends another FU response. On receiving a buffered data packet, the LPN can poll immediately to receive the next message.

Using a batteryless LPN, this communication becomes more complicated because the LPN might not always have sufficient stored energy to perform polling at a predefined polling interval, or immediately after receiving a buffered message. As the harvesting power can influence the polling interval, instead of a fixed predefined polling interval a batteryless LPN uses it as a minimum interval and waits longer to poll if not enough energy is available. Similarly, it will not poll immediately after receiving a buffered message, but only as soon as it has harvested enough power. During the process of receiving a buffered data packet, the LPN could experience multiple shutdown events as the required capacitor voltage to successfully receive a buffered data packet could be higher than its voltage at which it starts the communication. In simplicity, the LPN should start sending the FP only if it has acquired a sufficient threshold voltage ($V_{threshold}$). Initiating the poll from $V_{threshold}$, it can receive at least one buffered data packet successfully without reaching below the device turn-off voltage $V_{off}$. Thus, the overall DL latency for a batteryless LPN could increase (wrt. battery-powered) as the packets need to wait in the FQ until the LPN acquires the voltage $V_{threshold}$ and the poll interval expires. The LPN can be equipped with a hardware circuit, such as an ultra-low-power comparator with a power consumption in the order of pico-watts [31] to determine if it has reached $V_{threshold}$. The comparator can be configured to generate events every time the LPN voltage reaches $V_{threshold}$ or $V_{off}$. An UP event is generated whenever the LPN’s voltage reaches $V_{threshold}$ and a DOWN event whenever it falls below $V_{off}$ [32]. The LPN’s MCU can take appropriate actions based on these generated events. Using such an ultra-low-power comparator would not significantly impact the LPN voltage.

During the idle time, the LPN remains in sleep mode. In this time, the current consumption of the LPN includes the sleep mode current consumption of the MCU ($I_{sleepMCU}$) and main radio ($I_{sleepMR}$). The new voltage at the end of sleep mode can be predicted using Equation (3), where $I_{L}\left(\Delta t\right)=I_{sleepMCU}+I_{sleepMR}$. Before starting the communication, the comparator is used to compare the LPN instantaneous voltage with $V_{threshold}$. If that voltage is higher than the voltage $V_{threshold}$, the LPN initiates the events from top to bottom, as mentioned in Table 1, to receive the data packet from the FN. The LPN voltage changes according to the execution time and the current consumption of the corresponding events. $V_{threshold}$ is calculated by deducing the minimum initial voltage *V*(*t*) (using Equation (3)) by executing all the events from bottom to top mentioned in Table 1 starting with *V*(*t*+Δ*t*) equals $V_{off}$. The deduced initial voltage of each event needs to be above $V_{off}$.

### 3.4. WuR-Based LPN

**Figure 7:**
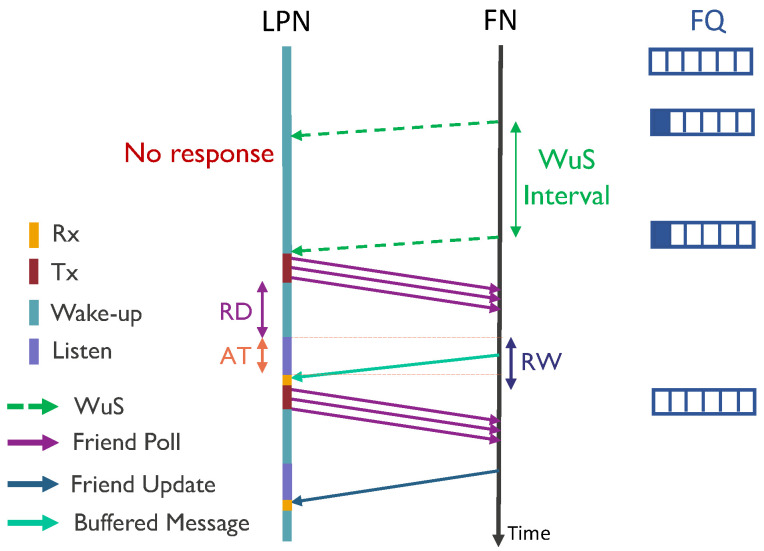
Sequence diagram to receive DL data packets using WuR-based communication.

The sequence diagram shown in Figure 7 presents the communication system to receive DL data packets by a WuR-based LPN. The WuR remains turned-on operating at orders of magnitude lower power consumption than regular radio while listening for a WuS [5]. This state where the LPN actively listens using the WuR keeping the main radio in deep sleep mode is named as wake-up state. When the FN receives a message for an LPN in an empty FQ, it initiates a communication event by sending a WuS. Upon receiving the WuS, the WuR can interrupt the main radio of the LPN to start the process to request the message. Similar to the poll-based LPN, to prevent the LPN from getting shut down during the communication, it wakes up the main radio to send the FP message only if it has the sufficient voltage ($V_{threshold}$). Thereafter, the LPN follows the same procedure as mentioned for the direct Poll scheme (cf., Section 3.3) by sending the FP messages in the three advertisement channels. To account for the potential loss of a WuS (e.g., because the LPN does not have enough energy to receive it or is temporarily shut down), the WuS is re-transmitted by the FN if no FP is received within a pre-configured WuS interval timer. While sleeping, the current consumption of a WuR-based LPN does not only includes the sum of the MCU sleep current $I_{sleepMCU}$ and main radio sleep current $I_{sleepMR}$, but also the WuR listening current $I_{listenWuR}$.

## 4. Results and System Analysis

This section presents the simulator setup and compares the performance of both considered LPN communication approaches, i.e., direct Poll- and WuR-based.

### 4.1. Simulation Setup

**Figure 8:**
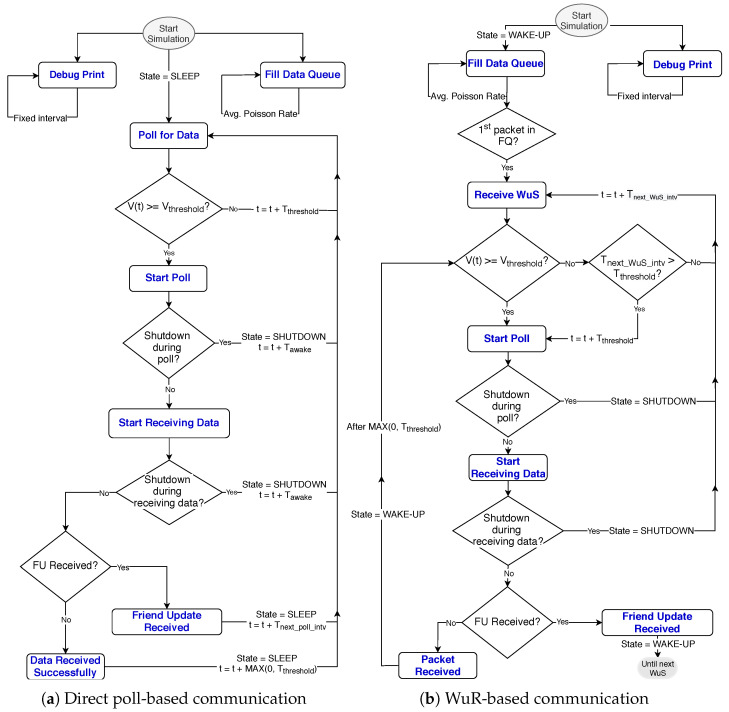
Flowchart of the simulator for batteryless LPN communication (V(t): Voltage of LPN at the time *t*, $T_{awake}$: The time after which LPN wakes up, $T_{threshold}$: The time to achieve $V_{threshold}$, $T_{next\_poll\_intv}$: The time of next polling activity, $T_{next\_WuS\_intv}$: The time of next WuS).

We implemented a Python-based simulator to imitate the friendship communication mechanism of the batteryless LPNs, as shown in Figure 6 and Figure 7. The simulator is capable of reproducing the BLE radio activities such as sending FP messages, and FQ buffered data packets, but also it implements the possibility of sending a WuS. The flow chart of the simulator is presented in Figure 8. Each experiment is run until a total of 25,000 packets have been generated according to a Poisson arrival process. To request and receive a buffered data packet, the LPN follows the sequence of events listed in Table 1, in the order from top to bottom. The table also mentions the execution time and the current consumption of the corresponding events for both types of LPNs (with and without a WuR). These time and current consumption values can be used as Δ*t* and $I_{L}\left(\Delta t\right)$ to calculate the LPN’s voltage at any time using Equation (3). The time and the current consumption of the main radio and the MCU are based on the Nordic nRF52 power profiler [33]. According to the datasheet of the AS3933 WuR [34], a WuR-based LPN consumes a current of 2.7 μA when one WuR channel actively listens to the incoming signals ($I_{listenWuR}$) and 12 μA while receiving them.

We consider a BLE data rate of 1 Mbps. Therefore, transferring a data packet of 68 B from an FN to an LPN needs 544 ms as specified in Table 1 (Event: Scan Message), and for the FP message of 48 B, 384 ms is needed (Event: Tx). The data packet size is calculated by the sum of the network PDU, advertising data (AD) type (1 B), message length (1 B), preamble (1 B), access address (4 B) and CRC (3 B). The calculated buffered data packet, FU message and FP message sizes are mentioned in Table 2. Other parameters defined in Table 2 are used to compare the LPN communication schemes. As such, $V_{min}$ and E are taken considering regulated supply for external components of the Nordic nRF52 as 1.8 V and 3.3 V, respectively [32]. The following performance metrics are considered in the comparison between the friendship communication mechanisms:

- Packet delivery ratio (PDR): The ratio of packets successfully received by the LPN compared to the total reaching the FQ.
- Downlink packet latency: The average latency to receive a packet by the LPN from the time it arrives at the FQ.

In each experiment, we calculate the values of the capacitor size and signal (WuS/Poll) interval that achieve the minimum DL latency, while maintaining maximum PDR. For simplicity, continuous power harvesting is assumed. We have considered the packet loss only due to the LPN not having enough energy to receive the WuS or the packet, but not due to interference or collisions. There have been many studies evaluating the impact of interference on BLE under other technologies such as ZigBee, IEEE 802.11, and IEEE 802.15.4 [35,36,37]. Such interference causes the reception of erroneous packets, thereby affecting the DL latency. Collisions could happen when multiple LPNs are attached to an FN with a short advertisement interval or deployed near BLE mesh nodes. The percentage of packet collisions with an advertising interval of 500 ms and having 7 BLE nodes is less than 0.4% [38]. The batteryless LPNs (maximum 7) connected to a FN generally transmit or receive data at a much lower frequency than 500 ms. As a consequence, the PDR and DL latency of the LPNs would be negligibly affected by the presence of other nearby LPNs.

### 4.2. Minimum Harvesting Power

As the LPN consists of many energy consuming components, it is needed to know the minimum harvesting power at which the LPN can still charge its capacitor to the threshold voltage $V_{threshold}$ while in sleep mode (or WuR listening mode). Moreover, the harvested power needs to be enough to be able to complete at least one full polling cycle with a fully charged capacitor (i.e., $V_{threshold}$ needs to be lower than the maximum capacitor voltage), given a specific capacitance. The minimum harvesting power is calculated based on Equation (3), where the final voltage *V*(*t* + Δ*t*) equals $V_{threshold}$ for the limit of Δ*t* towards infinity at sleep state. The harvesting power becomes independent of the initial voltage and the capacitor size, as for an infinitely large Δ*t*, the final voltage becomes $\left(E\cdot P_{h}\right)/\left(P_{h}+E\cdot I_{L}\left(\Delta t\right)\right)$. However, to perform the BLE friendship communication cycle (including sending a poll and receiving the response), different capacitor sized LPNs need a different threshold voltage. Accordingly, the required minimum harvesting power for different capacitance varies.

**Figure 9:**
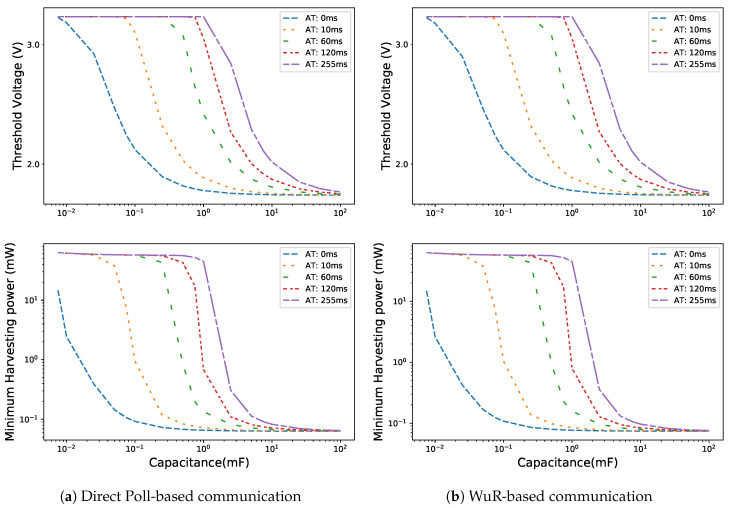
Threshold voltage and minimum harvesting power for different arrival times (ATs).

Considering the turn-off voltage $V_{off}$ of 1.8 V, the threshold voltage and corresponding minimum harvesting power for different ATs are shown in Figure 9. The minimum harvesting power does not vary much for the capacitor of 50 mF and higher. It is around 63.2 μW for the LPN with only a main radio and around 73.9 μW for the LPN with a WuR along with the main radio for such large capacitors. Both the communication schemes presented for the LPN (with and without WuR) show similar behaviour for the change in the values of minimum harvesting power and threshold voltage for different capacitor sizes. As the LPN with WuR has higher current consumption, it requires more harvesting power and the differences in the minimum harvesting power observed are up to 165.6 μW. Additionally, with the differences in the minimum harvesting power, the LPN with WuR has lower threshold voltage with the difference compared to the LPN without WuR up to 0.028 V. In the WuR enabled LPN (cf. Figure 9b) at AT equal to 0 ms the minimum harvesting power decreases exponentially from 14.8 to 0.077 mW for a capacitor size between 7.5 and 750 μF. However, with the increase in AT, this exponential decrease shifts to larger capacitor sizes. As with the increase in AT, the listening time of the LPN increases, which increases its energy consumption. Therefore, high threshold voltages are required for smaller capacitor sizes resulting in the increase of the minimum harvesting power needed.

### 4.3. WuR-Based and Direct Poll-Based Friendship Protocol Performance

**Figure 10:**
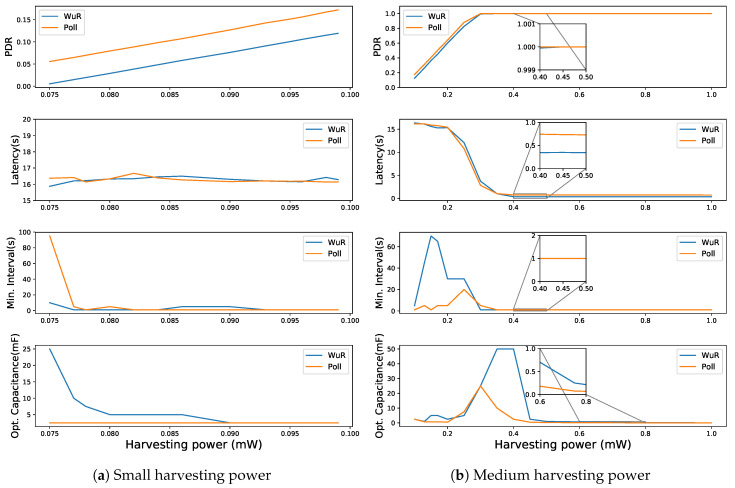
Comparing WuR-based and direct poll-based communication schemes for a Poisson packet arrival rate = 1 s.

**Figure 11:**
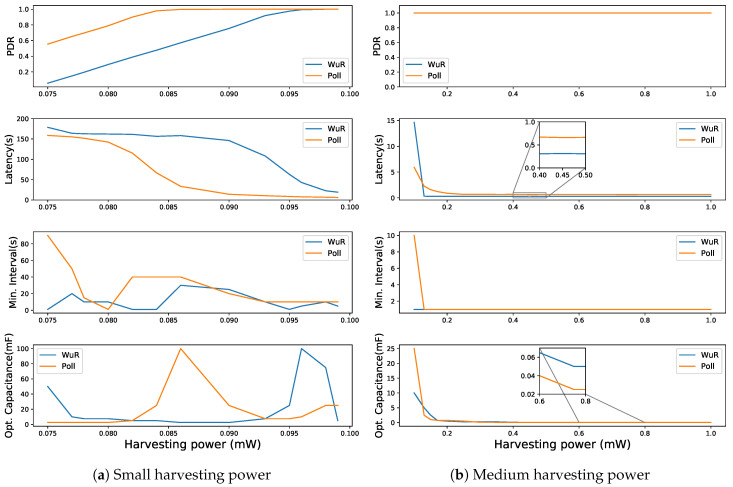
Comparing WuR-based and direct poll-based communication schemes for a Poisson packet arrival rate = 10 s.

**Figure 12:**
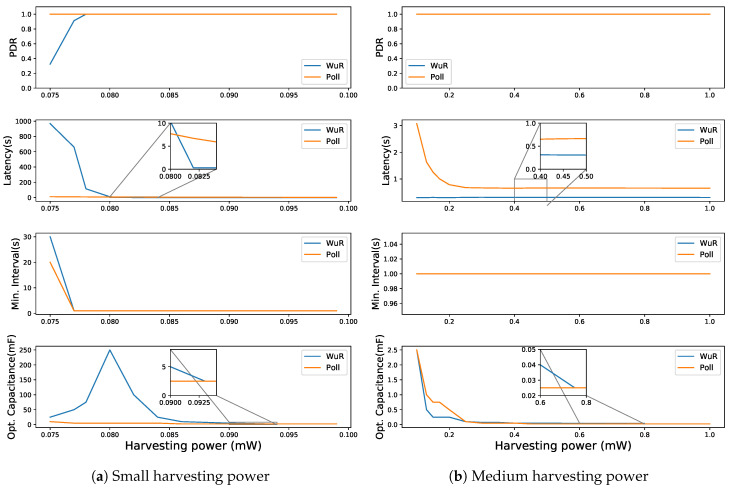
Comparing WuR-based and direct poll-based communication schemes for a Poisson packet arrival rate = 60 s.

The results are grouped based on the capabilities of different harvesting techniques which are small (0.075 to 0.099 mW) representing harvesting at the rate of electromagnetic or piezoelectric harvesting techniques, medium (0.1 to 1 mW) in line with indoor light, and large (1.1 to 500 mW) in line with techniques based on direct sunlight, mechanical movements, or thermal energy [39]. The parameters used to compare the performance of both the LPN communication schemes (WuR-based and direct Poll-based) are defined in Table 2. Generally, for a fixed harvesting power, with an increase in the signal (poll/WuS) interval (SI) values, the PDR decreases because the LPN polling frequency or receiving WuS notification frequency decreases. Furthermore, with the increase in the capacitance, the LPN can store more energy, and thus the PDR improves. Moreover, for a harvesting power, there exists a minimum optimal capacitance. More than that, further increasing the capacitor size above that value does not affect the PDR nor the DL latency. Therefore, for each value of harvesting power, we calculate this optimal capacitance and the minimum SI that can provide the highest PDR and lowest DL latency. We consider all capacitance and SI combinations that deviate at most 5% of the maximum achievable PDR and lowest DL latency as being optimal as well. This 5% deviation allows to eliminate minor differences in the PDR and the DL latency that occurs for the optimal and higher capacitance values due to the randomness in Poisson packet arrivals at the FQ. By allowing this 5% deviation, we can smooth the curves of optimal capacitance and interval. A value smaller than 5% did not provide the necessary smoothing effect, while a larger value would affect the optimality too much. Figure 10, Figure 11 and Figure 12 compare both the communication schemes for a Poisson packet arrival rate of 1, 10 and 60 s, respectively, showing the optimal capacitance and its corresponding PDR, DL latency value and minimum SI at different harvesting power values.

At the Poisson packet arrival rate of 1 s and for small harvesting power values (cf. Figure 10a), with the increase in the harvesting power, the PDR improves, but the DL latency does not. This is because, with the increase in harvesting power, the LPN can reach the threshold voltage faster, and so it polls to receive the data more frequently. The DL latency values do not vary at these harvesting powers because most of the packets are dropped from the FQ due to the low PDR. The observed variations in the DL latency values are due to the randomness in the Poisson packet arrivals. Moreover, as with the increase in harvesting power, the capacitor charges faster, and therefore, the optimal capacitance decreases. The minimum SI does not vary much at low harvesting power because the FQ is never empty and the LPN never gets an FU, and so the SI does not play any role. The WuR-based LPN achieves lower PDR values because some of its energy is wasted in listening to the periodic WuS while remaining in sleep mode and this delays acquiring the voltage $V_{threshold}$ to start the communication. With the increase in the delay to poll, more packets are dropped from the FQ. The FN sends these WuSs assuming the previous WuS has not reached the LPN (WuS might be lost in transmission or the LPN might be shutdown) as it does not get any response from the LPN. Therefore, the optimal capacitance required for the WuR-based LPN is also higher. Figure 10a shows that for low harvesting power (between 75 and 300 μW), direct Poll-based communication performs better, but neither approach achieves sufficiently high PDR. Whereas, above 300 μW both types of communication achieve the maximum PDR (cf. Figure 10b) and WuR-based communication starts to outperform direct Poll-based data communication in terms of DL latency. At the higher harvesting power values, when the PDR of around 80% is achieved (200 μW and above in Figure 10b), it is observed that with the increase in the harvesting power, the DL latency decreases and stabilizes to around 0.33 and 0.73 s for the WuR- and Poll-based approaches, respectively. With the increased value of the harvesting power, the LPN takes less time to achieve the threshold voltage. Thus, the DL latency is reduced with an increase in the harvesting power as seen from Figure 10b that it decreases drastically from 15.3 to 0.34 s for the WuR-based communication by increasing the harvesting power from 0.2 to 0.4 mW. The optimal capacitance is larger for the harvesting power at which the maximum PDR is achieved (such as at 400 μW in Figure 10b, at 86 μW in Figure 11a or 80 μW in Figure 12a), and the LPN can support frequent SIs. It means the LPN can harvest enough energy to successfully receive the WuS whenever they are sent and can frequently poll without letting the FN drop packets from the FQ.

At the Poisson packet arrival rate of 10 s for low harvesting powers, the PDR improves as compared to that of 1 s packet arrival rate, but the DL latency deteriorates (up to 178.6 s) as shown in Figure 11a. Similarly, it increases up to 967.67 s for a Poisson packet arrival rate of 60 s (cf. Figure 12a). As for the low Poisson packet arrival rate (1 s) and low harvesting power, a greater number of packets enter the FQ without being polled by the LPN and thus a greater number of packets are dropped due to the queue being full. However, as the Poisson packet arrival rate increases, a smaller number of packets are dropped, improving the PDR but also increasing the DL latency. With the higher Poisson packet arrival rate, the LPN receives the older packets that have waited longer, whereas low Poisson packet arrival rate drops the older packets and the LPN receives the recently added FQ packets obtaining lower DL latency. Moreover, at the Poisson packet arrival rate of 10 s, when the PDR of around 80% is achieved (80 μW and above for poll-based or 90 μW and above for WuR-based in Figure 11a), with the increase in the harvesting power, the DL latency starts decreasing drastically. For poll-based communication, it decreases from 142.3 to 8.6 s by increasing the harvesting power from 80 to 95 μW and for WuR-based communication, it decreases from 146.2 to 19.6 s by increasing the harvesting power from 90 to 99 μW. For higher harvesting power, as shown in Figure 11a, the latency of the WuR-based communication decreased lower than the poll-based communication but requires higher optimal capacitance.

Similar to the Poisson packet arrival rate of 1 and 10 s, at 60 s for WuR-based communication having a PDR of 80% or above (cf. Figure 12a) shows a decrease in the DL latency from 660.7 to 0.30 s with the increase in harvesting power from 77 to 82 μW. WuR-based communication performs better in terms of DL latency for the harvesting power above 82 μW where it achieve the DL latency of 0.30 s. For poll-based communication, the latency decreases to 0.66 s, but at a much higher harvesting power of 300 μW. Moreover, for Poisson packet arrival rates higher than 60 s, the conclusions are similar to that of 60 s showing improvements in PDR at lower harvesting power values for WuR-based communication.

Analysing the results for a harvesting power higher than 1 mW (graphs omitted), it is observed that the optimal capacitance for both the types of communication becomes the same at a high harvesting power. The value of harvesting power at which the optimal capacitance becomes the same decreases with an increase in the Poisson packet arrival rate. Moreover, with high harvesting power values, the DL latency values for both scenarios remain almost constant, where the WuR-based communication continues to perform better than direct Polling. WuR-based communication obtains 33.55, 30.26 and 30.21 ms of DL latency for the Poisson packet arrival rate of 1, 10, and 60 s, respectively; whereas, poll-based communication obtains 71.77, 65.42, and 64.88 ms.

It can be concluded that the poll-based data communication performs better for low power harvesting techniques such as electromagnetic or piezoelectric and for low irradiance indoor light (producing up to 400 μW power). In contrast, WuR-based data communication outperforms for medium and large harvesting power techniques. For high harvesting powers (e.g., using thermal energy), a small capacitor of only 50 μF can support all data rates (1 s and above) at maximum PDR. For medium harvesting power, a capacitance of 25 mF is enough to support a packet arrival rate of 10 s or more with a DL latency of maximum 5.9 and 14.64 s for poll- and WuR-based communication, respectively. To support high packet arrival rates of 1 s achieving maximum PDR, a larger capacitor of at least 50 mF is required. Finally, for a low harvesting power, a 100 mF capacitor is required to support a packet arrival rate of 10 s at maximum PDR.

## 5. Conclusions and Future Work

In this article, we studied the optimal parameters to perform the communication between a friend node and a batteryless low power node in BLE mesh networks. We studied the achievable PDR and latency of DL packets, considering different parameters (i.e., capacitance, energy harvesting power and Poisson packet arrival rate). The results have proven that a batteryless BLE device can easily support DL communications by using the BLE friendship feature, both using the traditional polling-based technique, or by employing a WuR. Even with harvesting power ranges in the order of tens of micro-watts, a packet arrival rate of 10 s can be supported without any packet loss. The WuR-based approach is mainly beneficial in terms of DL latency when the packet arrival rate is very low (i.e., 1 s) or high (i.e., 60 s). In these scenarios, it provides a DL latency reduction of more than 50% compared to the polling-based technique, that is from 71.77 to 33.55 ms at 1 s packet arrival rate. In summary, this work can be used to know the minimum harvesting power and the optimal capacitor size which can provide the desired PDR and DL latency for different configurations of the batteryless LPN and FN.

There are several future research directions. In our experiments, we have considered fixed values of the signal interval which can be optimised dynamically depending on the harvested power. As mentioned earlier, multiple low power nodes should be attached to a friend node to evaluate the impact of the collisions, and the interference due to the presence of other technologies. Moreover, the effect of the friend queue size and the impact on the power consumption of the friend node are interesting open research directions. As currently, the simulated results are presented, it is needed to perform experiments using real hardware. We are currently working on a hardware setup to validate the presented results.

## Figures and Tables

**Table 1:** Event sequence for a poll request and response of an LPN [32,33].

Event	Time [μs]	Current cons.	Current cons.	Comments
		of the LPN	of the LPN	
		without WuR	with WuR	
		[mA]	[mA]	
Rx WuS	60	N/A	0.81	Receive WuS
Radio wake-up	1510	0.798	0.8007	Prepare for poll transmission
TX (at 4 dBm)	384	11.75	11.7527	Tx advertisement packet on channel 37
Change channel	300	4.078	4.0807	Change radio frequency
TX (at 4 dBm)	384	12.15	12.1527	Tx advertisement packet on channel 38.
Change channel	330	4.328	4.3307	Change again the radio frequency
TX (at 4 dBm)	384	12.01	12.0127	Tx advertisement packet on channel 39.
Radio off	290	5.898	5.9007	Main radio turned-off
Post processing	620	1.618	1.6207	
Cool down	14,000	0.492	0.4947	Prepare to switch to the sleep state
Sleep	RD	0.01597	0.01867	Device in sleep mode
Wake-up prescan	1570	0.758	0.7607	Wake-up for Rx
Listen	AT	14.318	14.3207	Actively Listen for incoming message
Scan message	544 (or 464)	17.2	17.2027	Rx 68 B FQ data (or 58 bytes FU)
Radio off	320	6.858	6.8607	Turns off the main radio
Post-processing	26,300	0.488	0.48827	Set up the sleep timer for the next event
and Cool Down				and switch to sleep state

**Table 2:** Simulation parameters.

Parameters	Symbol	Value
Poisson packet arrival rate	$\mu_{p}$	{1, 10, 30, 60, 120} s
Harvesting power	$P_{h}$	[0.075, 200] mW
Capacitor size	*C*	[[0.0075, 500] mF
Signal (WuS/Poll) interval	SI	[1, 150] s
Turn-off voltage	$V_{off}$	1.8 V
Max operating voltage	*E*	3.3 V
Friend Queue size	$N_{fq}$	16 packets
Receive Delay	RD	255 ms
Arrival time (max up to RW)	AT	0 ms
Friend Queue message packet size	$N_{fqm}$	68 bytes
Friend Update packet size	$N_{fu}$	58 bytes
Friend Poll packet size	$N_{fp}$	48 bytes
